# A Deep Learning Framework for Classification of Neuroendocrine Neoplasm Whole Slide Images

**DOI:** 10.3390/cancers17182991

**Published:** 2025-09-13

**Authors:** Amir Hadjifaradji, Michael Diaz-Stewart, Jenny Chu, David Farnell, David Schaeffer, Hossein Farahani, Ali Bashashati, Jonathan M. Loree

**Affiliations:** 1 School of Biomedical Engineering, University of British Columbia, Vancouver, BC V6T 2B9, Canada; ahadjifaradji@gmail.com (A.H.); michael.diaz@ubc.ca (M.D.-S.); h.farahani@ubc.ca (H.F.); 2 Department of Pathology and Laboratory Medicine, University of British Columbia, Vancouver, BC V6T 1Z7, Canada; jenny.chu@islandhealth.ca (J.C.); david.farnell@vch.ca (D.F.); david.schaeffer@vch.ca (D.S.); 3 Royal Jubilee Hospital, Victoria, BC V8R 1J8, Canada; 4 Vancouver General Hospital, Vancouver, BC V5Z 1N1, Canada; 5 Division of Medical Oncology, BC Cancer, Vancouver, BC V5Z 4E6, Canada

**Keywords:** neuroendocrine, deep learning, grading, object detection, mitotic figures

## Abstract

**Background/Objectives**: Neuroendocrine neoplasms (NENs) are uncommon neoplasms. Grading informs the prognosis and treatment decision of NENs and is determined by cell proliferation, which is measured by mitotic count and Ki-67 index. These measurements present challenges for pathologists as they suffer inter- and intra-observer variability and are cumbersome to quantify. To address these challenges, we developed a machine learning pipeline for identifying tumor areas, proliferating cells, and grading NENs. **Methods**: Our study includes 385 samples of gastroenteropancreatic NENs from across British Columbia with two stains (247 H&E and 138 Ki-67 images). Labels for these cases are at the patient-level, and there are 186 patients. We systematically investigated three settings for our study: H&E, H&E with Ki-67, and pathologist-reviewed and corrected cases. **Results**: Our H&E framework achieved a three-fold balanced accuracy of 77.5% in NEN grading. The H&E with Ki-67 framework yields a performance improvement to 83.0% on grading. We provide survival and multivariate analysis with a c-index of 0.65. Grade 1 NENs misclassified by the model were reviewed by a pathologist to assess reasons. Analysis of our AI-graded NENs for the subset of pathologist-assessed G1s demonstrated a significant (*p*-value = 0.007) survival difference amongst samples the algorithm assigned to a higher grade (*n* = 20; median survival 4.22 years) compared to concordant G1 samples (*n* = 60; median survival 10.13 years). **Conclusions**: Our model identifies NEN grades with high accuracy and identified some grade 1 tumors as prognostically unique, suggesting potential improvements to standard grading. Further studies are needed to determine if this discordant group is a different clinical entity.

## Simple Summary

Neuroendocrine tumors are uncommon cancers that use grade to guide management. Grading relies on cell counts of the number of cells undergoing mitosis and those staining positive for Ki67. This study developed a machine learning tool to automate grading by analyzing tissue images. The tool showed potential to identify patients with worse outcomes, even when their tumors appeared low grade. This could lead to better-informed treatment decisions.

## 1. Introduction

Neuroendocrine cells are specialized cells dispersed throughout the body. Uncommon neoplasms known as neuroendocrine neoplasms (NENs) may develop in these cells and commonly originate within the gastrointestinal (GI) tract, pancreas, and lungs [1,2,3,4]. Three key features that categorize NENs are the site of origin, differentiation, and grade. NEN differentiation is sub-categorized into well- and poorly differentiated neoplasms, also referred to as neuroendocrine tumors (NETs) and neuroendocrine carcinomas (NECs), respectively, and are morphologically distinct. NETs are heterogeneous tumors whose grades do not present morphologically distinct features, and that are assessed based on the proliferation of the tumor [1,5,6,]. This presents pathologists with several challenges when assessing NETs.

Clinicians utilize two measures for grading as follows: Mitotic count and Ki-67 index (see Table 1). Mitotic count is observed in H&E slides and is defined as the number of mitotic figures in 10 high power fields, representing approximately 2 mm^2^ of tissue under a microscope at 40× objective magnification. Ki-67 index is observed in Ki-67-stained slides and is defined as the proportion of Ki-67 positive NEN cells. Grading guides clinicians on treatment decisions and is prognostic. Higher grades are more likely to have metastatic disease and larger tumor size. The two measures can have discrepancies in which case the highest grade takes precedence [1,2,6,,7,8].

NENs, though relatively rare with an estimated incidence of 3–6 per 100,000 annually, have been increasing in prevalence over the past several decades [1,2,3]. Accurate grading carries significant clinical implications, as grade is strongly correlated with prognosis, risk of recurrence, and therapeutic decision-making. For example, lower-grade tumors may be managed with surveillance or somatostatin analogs, whereas higher-grade tumors may require chemotherapy or targeted agents as an initial therapy [5]. Thus, reliable and reproducible grading is essential for optimizing patient outcomes and guiding treatment pathways.

A challenging aspect of clinically assessing NETs is the heterogeneity of tumors. Heterogeneity, in this case, means that within different portions of the tumor, some areas have higher proliferation than others and identifying these hot spots can be difficult. Furthermore, identifying proliferating cells suffers inter- and intra-observer variability. Cells undergoing mitosis can resemble pyknotic nuclei, inflammatory cells, and hyperchromatic cells. The key morphological difference is jagged chromatin edges rather than smooth edges [9,10,11]. Ki-67 can result in gland cells and lymphocytes being immunopositive [12] and determining if a cell is immunopositive can be subjective.

Several recent studies have investigated the application of deep learning and digital pathology to the grading or prognostication of NENs, though most have focused on specific aspects such as Ki-67 quantification or imaging-based prediction, rather than offering a comprehensive grading framework. For example, automated analysis of Ki-67 immunohistochemistry on whole slide images has demonstrated strong concordance with manual assessment in pancreatic NENs, with one study reporting a Pearson correlation of 0.968 and 92.4% grade concordance using a commercial image analysis platform [13]. Similarly, other work has evaluated the reproducibility of Ki-67 index determination across digital platforms such as AI4Path.ca and QuPath, noting general agreement with manual methods but also emphasizing variability between tools and the challenges of standardization in clinical settings [14]. While these efforts support the feasibility of digital Ki-67 assessment, they are typically limited to immunohistochemistry and do not incorporate architectural or morphological features from H&E slides, nor do they evaluate downstream clinical outcomes.

Other related studies have explored broader applications of computational pathology to NENs and other tumor types, including prognostication and molecular subtyping. One study integrated deep learning-based radiomics from CT imaging with pathomic features derived from Ki-67 staining to predict postoperative liver metastasis in pancreatic NENs, achieving high predictive performance (AUC > 0.96) in internal validation [15]. Although promising, this model was developed for recurrence risk prediction and did not attempt histological grading. Beyond NENs, the DeepTFtyper model applied a graph neural network to infer molecular subtypes of small cell lung cancer from H&E images, highlighting the potential of context-aware deep learning to capture clinically relevant histologic patterns [16]. Additionally, the recently proposed OMG-Net introduced a generalizable two-stage pipeline for mitotic figure detection across multiple cancer types using H&E slides, with strong performance metrics (F1 = 0.84) but no integration of Ki-67 or application to tumor grading [17]. Compared to these approaches, our study combines H&E-based mitotic figure detection with automated Ki-67 quantification and introduces a histogram-based aggregation strategy that may better capture tumor heterogeneity.

There is great potential for digital pathology to aid pathologists by providing areas to focus on, reducing variability, and efficiently processing whole-slide images (WSIs). The current research aims to provide a standardized tool for evaluating NENs, which combats inter- and intra-observer variability, especially in low-volume centers. Through this, we may also alleviate some of the burden experienced in clinical practice and improve management of the disease or increase efficiency [18]. We developed a deep learning framework for grading NENs. The workflow was designed to emulate a pathologist’s workflow, while attempting to identify key features used for grading NENs.

## 2. Materials and Methods

### 2.1. Data Acquisition and Processing

The development of our NEN grading framework utilized two datasets. The first phase of development utilized MItosis DOmain Generalization 2022 (MIDOG22) [19], a publicly available dataset for mitotic detection, due to the availability of strongly annotated mitotic figure. MIDOG22 contains 354 annotated cases from 5 different tissues: 150 human breast cancer, 55 human neuroendocrine tumors, 50 canine cutaneous mast cell tumors, 55 canine lymphomas, and 44 canine lung cancers. The total number of annotations is 9501 mitotic figures. Phase two of development utilized our internal dataset which contained slide-level labeled tumor grades and non-exhaustively annotated tumor tissue. Our internal dataset comprised 385 samples from various centers across British Columbia, Canada, with two different stains, from both primary and metatstatic cancers. All samples are of resected tissue slides of which 247 are H&E and 138 are Ki-67 slides. Among the H&E resected tissues were 152 GI tract slides, 73 pancreas slides, and 22 unknown primary site slides. The total number of patients is 186, 102 of which were metastatic at diagnosis. Table 2 provides a breakdown of the dataset. All slides have been scanned with an Aperio AT2 slide scanner (Leica Biosystems, Nussloch, Germany) at 40× objective magnification, with resolutions of 0.2525 µm. Grade 3 NET and NEC cases are categorized into a single group for several reasons, primarily because there are very few NECs within our dataset. Despite the morphological differences, identification of mitotic figures and Ki-67 positive cells are similar for either morphology, as well as their WHO cut-offs [20,21]. Labels for our dataset are at a patient-level, with the highest grade, mitotic count, and Ki-67 index provided. Due to the lack of cell annotation or hot-spot annotation used in the assessment of the grade in our cohort, we utilize an external dataset for development of our mitotic figure detection algorithm.

For the purpose of training, the internal dataset was split into three cross-validation folds. This led to three cross-validation permutations where in each permutation, two folds are used for training (approx. 66% of the data), and the last fold is split into testing and validation data (approx. 17% of the data each). Folds were stratified by grade to ensure consistent class distribution. Balanced accuracy was used as the target metric to correct for data imbalance. Additionally, class-weighting was applied to ensure equal grade-wise importance during training. We report the average of these three folds.

### 2.2. Machine Learning-Based Analysis Workflow

**Figure 1:**
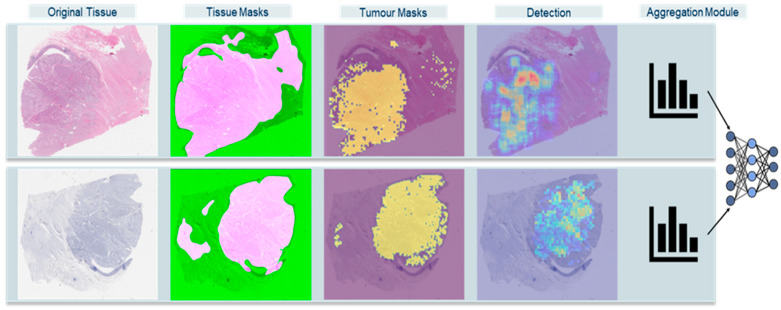
High-level overview of extended Ki-67 framework approach. Original Tissue: WSI thumbnail. Tissue Masks: HistoQC fused masks, pink areas are the usable tissue areas with minimal artifacts. Tumor Masks: identified tumor areas overlapped on WSI. Detection: Mitotic Activity and Ki-67 index density maps. Aggregation Module: histogram combined with machine learning classifications.

Figure 1 demonstrates an overview of our proposed framework.

#### 2.2.1. Tissue Masks

As a preprocessing step to our framework, we ran an open-source software, HistoQC (version 2.1) [22] for quality assurance of the dataset. The software generates a mask of usable tissue whereby any artifacts such as markers and blurry regions are excluded.

#### 2.2.2. Tumor Masks

The first step of our pipeline is to identify tumor regions in WSIs, as these are the areas used to assess tumor grade. A total of 80 WSIs were annotated by a pathologist to mark areas that contain a tumor. The 80 WSIs were divided into small patches, and we reduced the problem to patch-based classification to create these tumor segmentations by splitting the WSI into tumor and normal patches. The highest magnification of the digital slides was 40× and, with the 80 annotated WSIs, we extracted 71,659 PNG images (35,570 “Normal” and 36,089 “Tumor”) at the size of 1024 × 1024 pixel and downsampled the images to 512 × 512 pixel, giving patches an effective magnification of 20×. With these images, we proceeded to build a binary tumor-normal classifier with EfficientNet-B3 [23]. The following parameters were used to train the model: batch size of 8, trained for 27 epochs, used Adam optimizer with a learning rate of 5 × 10^−6^ with a weight decay of 0.0001 and amsgrad set to true.

#### 2.2.3. Mitotic Figures Detection

Using MIDOG22, we trained a variation in RetinaNet [24] introduced by Wilm et al. [25] regarded as RetinaNet-DA to detect mitotic figures from H&E images. RetinaNet-DA includes a domain adversary module in the feature extraction backbone which allows the network to extract domain-invariant features. RetinaNet was trained on over 100,000 patches of 512 × 512 pixel at 40× magnification with the following parameters: ResNet18 with ImageNet weights, all layers were trainable, the batch size of 20, learning rate of 0.0001, domain loss coefficient of 1, and trained for 200 epochs.

#### 2.2.4. Ki-67 Detection

Many studies for immunopositive cell detection [12,26] will train deep learning models on data where the ground truth has been generated by a traditional image processing approach, foregoing the need for manual annotation. As our end task is to grade NENs, we opt to calculate the Ki-67 index of Ki-67-stained patches using these traditional image processing approaches. Our algorithm was developed to evaluate fixed, non-overlapping patches of size 1536 × 1536 pixel, and only patches with a minimum of 500 cells. Patches were separated into a hematoxylin (H), eosin, and DAB (D) channels for which only the H- and D-channels were used. Each channel is then converted into a grayscale image whereby Otsu’s method is performed to generate a binary mask of cells. A 5-pixel expansion of the masks is applied, followed by Hough Circle transforms from OpenCV [27] to detect circles. To calculate the index of each patch, we divide the number of detected circles in the D-channel (positive cells) by the number of detected circles in the H-channel (all cells).

#### 2.2.5. Density Map Generation

Density maps of mitotic activity and Ki-67 index were generated for each slide. In H&E slides, we achieve this by quantifying the number of candidate mitotic figures detected by our object detection model for every 512 × 512 pixel patch. We then pass a 2 mm^2^ (5632 × 5632 pixel or 11 × 11, 512-pixel patches) filter through the quantized array to sum the number of mitotic figures in a 2 mm^2^ area. For Ki-67, density maps are simply the Ki-67 index calculated in each 1536 × 1536 pixel patch.

#### 2.2.6. Aggregation Modules

The small chunks of data used to process a WSI must eventually be aggregated in a manner that represents the slide- or patient-level. In our study, we evaluate three common aggregation techniques: (i) Majority Voting, (ii) Multi-Instance Learning, and (iii) Histogram of Density Maps. If two or more slides from the same patient have different grades, the higher grade is assigned to the patient).

**Figure 2:**
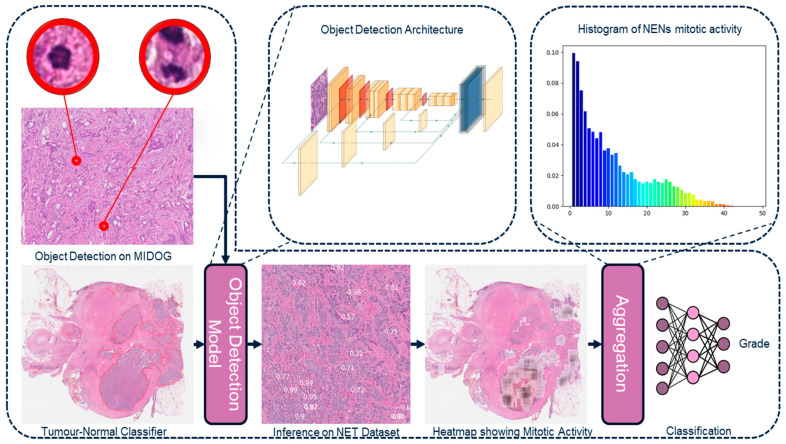
Overview of the histogram approach of the H&E framework.

(i) Majority Voting. Patches are considered independent from one another and are separately passed through a neural network for training. For inference, the neural network will try to classify the patch, and a final slide-level label is determined by taking the major predicted class for a slide. If patients have multiple slides, the slide with the highest grade is assigned the final grade.

(ii) Multi-Instance Learning (MIL). We extract features describing each patch using the KimiaNet [28] extractor and then apply an attention pooling mechanism to reduce the features to a single vector for classification. We employed three different MIL models for our study and adopted the default architectures and hyperparameters from their original implementations. For DeepMIL, we utilized an attention-based pooling mechanism with a LeNet-5-style architecture as the feature extractor, training with the Adam optimizer and a learning rate of 0.001 [29,30]. VarMIL extends DeepMIL by incorporating an attention-weighted variance module to better represent tissue heterogeneity, and we retained the same underlying settings [31]. The NoisyAnd-MIL model employed a Noisy-AND pooling function, which is designed to be robust to outliers, particularly in scenarios with a large number of instances [32]. We used the 2 mm^2^ area with the highest mitotic count as the input to the MIL models.

(iii) Histogram of Density Maps. The histogram represents cell proliferation, mitotic activity, or Ki-67 index, across a slide. The H&E slide’s histogram values range from 0 to 48, for a total of 49 bins and the frequencies are normalized. There were 6 cases that exceeded a mitotic count of 48, the array with the density maps for these cases were clipped to 48 because it is well above the G3 threshold. Figure 2 highlights the H&E pipeline which processes slides with the mitotic detection model and the histogram of density maps to predict grades. The histogram of Ki-67 slide values ranged from 0 to 100. These histograms were used as inputs to the neural network. The motivation for using histograms was to have a model, such as the Multi-Layer Perceptron (MLP) or logistical regression, learn complicated patterns and distributions that may account for the heterogeneity of the tumor.

#### 2.2.7. Statistical Methodology

Survival analysis was performed using the Lifelines Python library (version 0.27.8), which supports right-censored data by default [33]. Kaplan–Meier (KM) curves were generated with 95% confidence intervals to visualize survival probabilities over time. For multivariate analysis, we employed the Cox proportional hazards model implemented by Lifelines. The model’s assumption of proportional hazards was tested using built-in diagnostics (Schoenfeld residuals). The most commonly used measure of c-index is Harrel’s c-index, which is what we have used. The measure is derived from the Cox regression model and measures the rank correlation of the model’s predictions with the survival times. The c-index is the ratio of concordant pairs and total possible pairs [34,35].

## 3. Results

### 3.1. NEN Grading

Table 3 summarizes our three-fold average results for our experiments with H&E slides and with the addition of Ki-67 slides. Firstly, we investigated the results of our framework solely on the H&E component, to determine the baseline and the necessity of processing Ki-67. Using H&E stained WSI, we observed that the results for patch-based aggregation, and MIL-based aggregation are very poor. However, our approach utilizing the detection and histogram of mitotic activity with an MLP classifier yielded the best results and achieved a 3-fold balanced accuracy of 77.5%.

Once we determined that the histogram of mitotic activity yielded the best performance, our next investigation was to determine the added benefits of processing Ki-67 to our deep learning pipeline. When incorporating Ki-67, we investigated four possible aggregations, two naïve approaches which treat the two stains as separate pipelines, and two concatenated approaches which combine the histograms from both stains. “Naïve Approach 1” takes the max grade from the grade predicted by H&E neural network and Ki-67 index as defined by WHO. “Naive Approach 2” takes the max grade from the predictions of a Ki-67 neural network and the H&E neural network. Both naive approaches improve results in our three-fold balanced accuracy by 4.6%. The best model for the three-fold average balanced accuracy was the concatenated feature MLP. This model concatenates the generated histograms from RetinaNet-DA mitotic activity, and the Ki-67 detections into a single vector and trains a single neural network. This model achieved a 3-fold balanced accuracy of 83.0%. The performance improved by 5.5% when compared to the performance of the previous.

Finally, we re-examined the misclassified cases with an expert NET pathologist who provided insight into possible reasons for their misclassification. Seven misclassified cases were attributed to poor segmentation masks from the tumor-normal classifier. These segmentations included reactive, liver, or epithelial with hotspots detected in these areas. Furthermore, two cases were clinically misattributed with a grade 1 label and later deemed as G2s. This pathologist-guided examination allowed us to correct these cases by adjusting the tumor masks and correcting the grading labels. Results for the corrections on the full cohort are noted and can be seen in Table 4 and Table 5.

**Table 4:** Classification metrics with 95% confidence intervals for our framework and corrections.

Source	Method	Precision	Recall	F1 Score
H&E	Histogram MLP	0.667 (0.590, 0.741)	0.719 (0.646, 0.787)	0.686 (0.611, 0.756)
H&E + Ki67	Naïve Combination 1	0.695 (0.620, 0.769)	0.784 (0.732, 0.832)	0.721 (0.650, 0.790)
	Naïve Combination 2	0.690 (0.618, 0.763)	0.781 (0.729, 0.832)	0.718 (0.647, 0.784)
	MLP Concatenated Features	0.759 (0.689, 0.826)	0.757 (0.686, 0.824)	0.756 (0.690, 0.818)
H&E + Ki67 (Corrected)	Naïve Combination 1	0.730 (0.654, 0.804)	0.811 (0.759, 0.857)	0.757 (0.689, 0.825)
	Naïve Combination 2	0.721 (0.649, 0.796)	0.803 (0.749, 0.850)	0.749 (0.681, 0.813)
H&E	Pathologist Grade	0.924 (0.886, 0.955)	0.810 (0.732, 0.880)	0.849 (0.774, 0.910)
H&E + Ki67	Pathologist Grade (Ground Truth)	1 (1, 1)	1 (1, 1)	1 (1, 1)

**Table 5:** Summarized survival with our framework and corrections.

Source	Method	c-Index	Median Survival (yrs)		
			**G1**	**G2**	**G3**
**H&E**	**Histogram MLP**	0.63	7.20	4.88	1.74
**H&E + Ki67**	**Naïve Combination 1**	0.63	6.85	4.88	1.74
	**Naïve Combination 2**	0.65	7.78	4.56	2.10
	**MLP Concatenated Features**	0.63	6.77	4.63	1.22
**H&E + Ki67** **(Corrected)**	**Naïve Combination 1**	0.63	7.20	4.88	1.74
	**Naïve Combination 2**	0.65	7.50	4.56	1.74
**H&E**	**Pathologist Grade**	0.63	6.86	4.56	0.99
**H&E + Ki67**	**Pathologist Grade**	0.64	7.20	4.88	1.22

### 3.2. Survival Results

**Figure 3:**
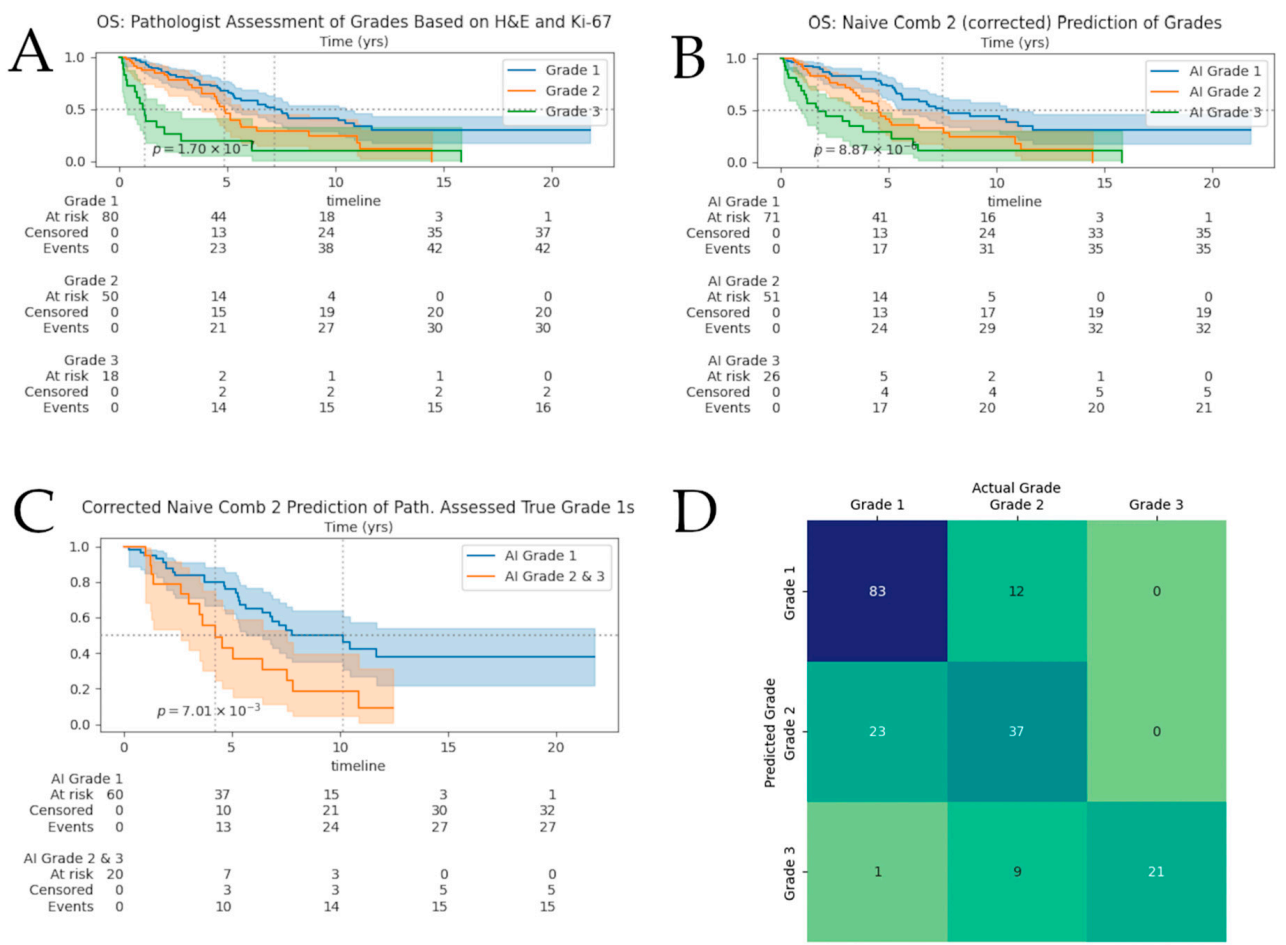
Survival analyses and grade-prediction confusion matrix. Shaded areas surrounding survival curves represent 95% confidence intervals. At-risk tables broken-down by grade can be found under the corresponding Kaplan–Meier curves. (**A**) Survival of NENs: pathologist-assessed grades based on H&E and Ki-67; (**B**) Kaplan–Meier curves for our extended framework; (**C**) Kaplan–Meier curves for AI-predicted grades of pathologist-assessed G1s; (**D**) Confusion matrices for our framework on the full cohort. Numbers displayed are absolute counts.

Kaplan–Meier (KM) curves for our computer-graded groups and pathologist-assessed grades can be found in Figure 3. The curves exhibit similar results with significant *p*-value. We observed that our “naive combination 2” framework was able to stratify patients with a c-index of 0.65. The c-index for these measures were slightly above the c-index from the pathologist’s true grade assessment, which was 0.64.

Figure 3D shows the confusion matrix for our full-cohort after pathologist-guided correction of tumor-masks and labels. We observed roughly 20% of cases were given a higher grade than the ground truth; as a result, we investigated further the KM curves for the pathologist-assessed true G1s and our framework predicted grades of these G1s in Figure 3C. We observe that the “Naïve Approach 2” showed significant separation between the computer-graded correctly classified G1s and computer-graded misclassified G1s; the median survival for each computer-graded group was 10.13 (*n* = 60) and 4.22 (*n* = 20) years, respectively. There was no significant separation found for AI-assessed G1s of pathologist-assessed true G2s.

### 3.3. Multivariate Analysis

**Figure 4:**
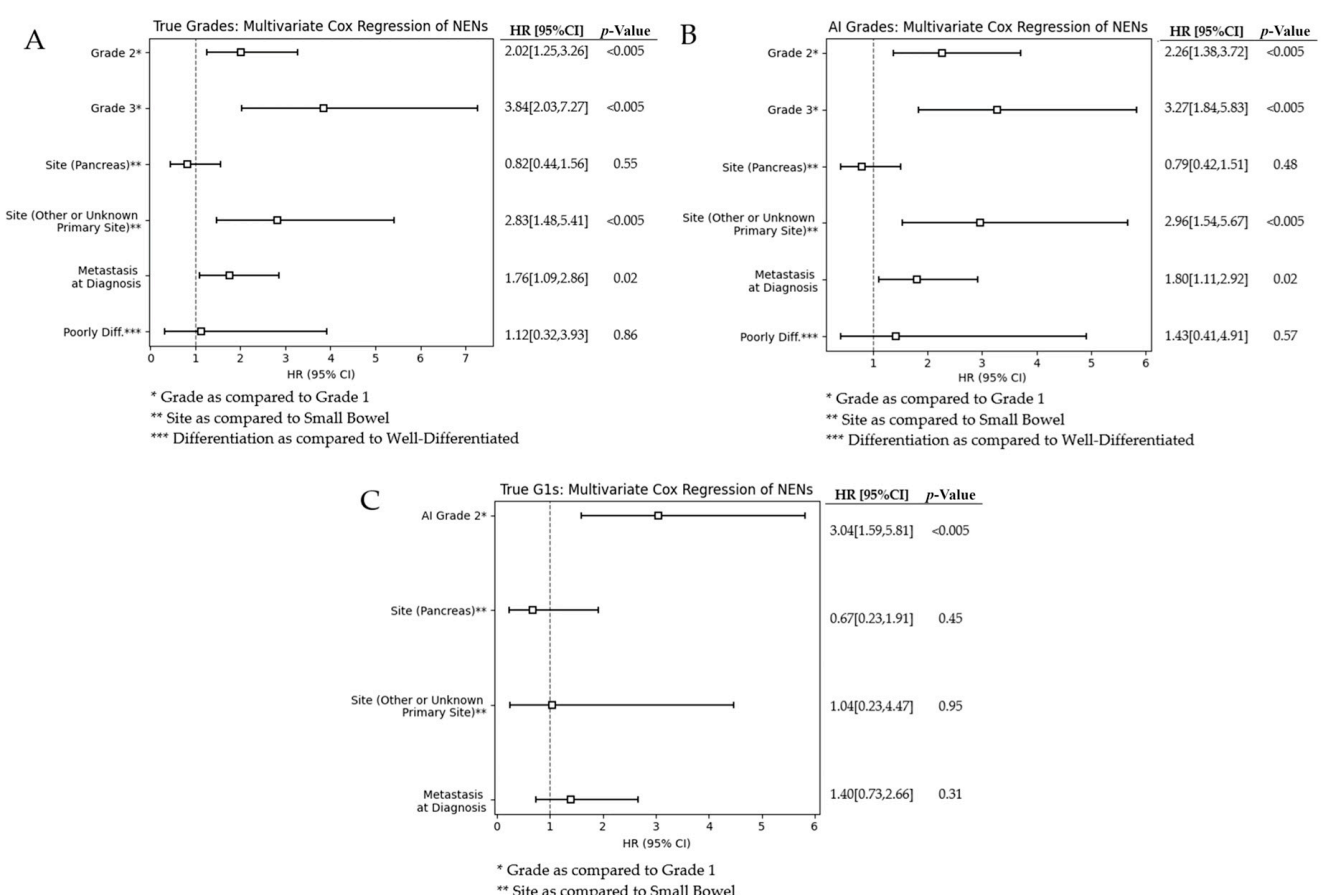
(**A**) Multivariate analysis of pathologist-true NEN grades; (**B**) Multivariate analysis of our framework-graded NENs; (**C**) Forest plot of AI-graded true G1s.

We investigate the confounding effects of various factors by performing a multivariate analysis on grades, sites, differentiation, and metastasis at diagnosis. Figure 4 shows the forest plots for our multivariate analysis. The figures demonstrate our multivariate analysis for pathologist-graded NENs, AI-graded NENs, and AI-graded G1s. Grade 2 and 3 NENs are compared to the baseline hazard of G1s, and the pancreas and other primary sites are compared to the baseline hazard of the small bowel. We observed that the hazard ratios (HRs) for all covariates between pathologist-graded and AI-graded cases are very similar. The largest HRs are G2s, G3s, and other or unknown primary sites. Grading is known for providing prognostic information; thus, it was expected it would have a relatively large HR. We believe that the similar values between AI- and pathologist-graded NENs demonstrate our network’s ability to grade and stratify groups.

The bottom forest plot in Figure 4 shows our computer-grades of pathologist-assessed true G1s. Neural network decision-making is not always intuitive and can sometimes use factors outside of what we may believe is driving predictive capabilities. Thus, it is imperative that we investigate what known factors are affecting the survival of this subset group. We observed that metastasis at diagnosis and the different sites of origin have a lower hazard ratio than either of the AI-misclassified G1s.

## 4. Discussion

Deep learning and digital pathology are rapidly evolving with substantial advances in diagnostics, prognostics, and discovery. Our research continues to merge these two fields to assist pathologists in NEN grading. Previous studies applying deep learning to NENs have largely focused on Ki-67 quantification due to its clinical relevance and well-defined thresholds [36]. These models have demonstrated high concordance with pathologist assessments (ICC ≈ 0.89) and highlight the promise of AI in reducing observer variability. Moreover, digital image analysis of Ki-67 in pancreatic NENs has been shown to achieve near-manual accuracy (coefficient ≈ 0.94), though stromal misidentification and staining variability remain challenges [37]. Our study extends this body of work by integrating both H&E and Ki-67 modalities into a unified deep learning framework that emulates the pathologist’s workflow.

A central design consideration in our framework is the method of aggregating local features into slide-level or patient-level predictions. The majority voting approach serves as a simple baseline, whereas MIL is a robust approach designed for weakly supervised settings, operating under the assumption that one or more informative patches are sufficient to determine the class label. We observed that histogram-based aggregation outperformed patch-based and MIL methods, supporting recent findings that tumor-level statistical summaries often better capture histopathological heterogeneity than local features [38].

This improved performance highlights a key insight: local patch-wise analysis, especially in heterogeneous tumors like NETs, often lacks sufficient spatial context for accurate grading. While MIL frameworks assume that a single discriminative patch can determine a slide-level label, this assumption fails in NEN grading, where proliferation must be assessed over standardized tissue areas (e.g., 2 mm^2^). Recent efforts to capture Ki-67 heterogeneity spatially have shown improved biological relevance in GEP-NENs, further justifying our approach of using full-slide statistical distributions [39].

Based on our results and the biology of NENs, we infer that the poor performance of majority voting and MIL-based methods is due to both the inherent heterogeneity of these tumors and the small field of view in patch-based models. A 512 × 512 pixel patch at 40× magnification covers less than 1/100th of the required 2 mm^2^ area for grading, providing insufficient signal. This limitation is further exacerbated in MIL models, which rely on the presence of a few highly informative patches. Our histogram-based method addresses this by capturing the global distribution of proliferative activity, consistent with recommendations from the College of American Pathologists to sample over 10 mm^2^ and compute the average across high-density areas [15]. This strategy also aligns with emerging computational methods like HipoMap, which emphasize spatially aware histological representation [38].

From a translational perspective, the clinical deployment of deep learning models presents both opportunities and barriers. Reliable deployment depends on high-quality WSIs, standardized staining, and access to computational resources. These conditions that may not be universally met across institutions. Regulatory approval and clinician acceptance will also require transparency and interpretability, which remains a key challenge in deep learning. While our model is not inherently explainable in a rule-based sense, the use of histograms and heatmaps provides a middle ground that supports visual inspection and clinical reasoning [40]. Simplifying the model into a rule-based system or logistic regression on handcrafted features may be feasible using histogram bins but would likely result in a trade-off in predictive performance.

Another promising frontier is the integration of radiomics or combined imaging modalities with histopathology. Integrating these imaging biomarkers into our histogram-based deep learning framework could provide a more comprehensive assessment of tumor biology and may streamline preoperative decision-making. For example, research has shown that endoscopic ultrasonography (EUS) allows high-resolution visualization of pancreatic and gastrointestinal NETs, improving tumor delineation and prognostication when combined with radiomic texture features extracted from CT, MRI, or elastography images [41]. Similarly, contrast-enhanced ultrasonography (CEUS) of liver metastases can quantify dynamic vascular properties, such as arterial enhancement and wash-out kinetics, that have been shown to correlate with Ki-67 proliferation indices, suggesting their utility in non-invasive tumor profiling [42]. Exploring these avenues was unfortunately beyond the scope of this study as cross sectional images were not available for incorporation.

Several other limitations must also be acknowledged. First, our model was trained on a dataset from British Columbia, which may limit generalizability to other regions or institutions. Although the cohort is diverse in geography and clinical practice, global variability in grading standards, staining protocols, and scanner types could affect model performance. External validation on international datasets is essential for robustness. Second, although our framework improves interpretability through visual summaries, the underlying deep learning architectures remain largely opaque, making it difficult to fully explain model decisions to clinicians. Additionally, while we have taken effort to combat data imbalance through the use of class-weighting and balanced accuracy as a target metric, additional exploration into sophisticated methods such as resampling or ensemble strategies could lead to improved performance.

Furthermore, tumor pathology assessment does not rely solely on mitotic count and Ki-67 index. Important prognostic markers such as necrosis, vascular invasion, and molecular alterations were not included in our model. Future work should consider incorporating these data streams using multimodal AI approaches, instead of just focusing on grade. Lastly, real-world implementation of such a system would require robust preprocessing pipelines, reliable tumor segmentation, and a pathologist-in-the-loop design to mitigate errors, such as those which we encountered during our pathologist review.

Ultimately, our results demonstrate the potential for deep learning to support and augment traditional pathology workflows. However, successful clinical translation will depend on addressing these feasibility and interpretability barriers and validating generalizability across broader patient populations and clinical settings.

## 5. Conclusions

In conclusion, using histograms of proliferating cell markers can produce good slide-level representations and yield high performing models. In our study, we demonstrated that our framework achieved an average balanced accuracy of approximately 83%. Our survival analysis with pathologist-guided input demonstrated that AI grades have a c-index of 0.65, and each grading group was separated with a significant *p*-value (*p*-value = 8.9 × 10^−6^). There exists some separation which is significant amongst the G1 subset, whereby AI over-graded cases present a lower median survival. While this framework is a promising step toward combating inter- and intra-observer variability in NEN grading, these experiments require further validation of other cohorts. From a clinical perspective, the over-graded G1 subset highlights an interesting potential for other factors to affect the stratification or grading of NENs and if validated, this may ultimately allow clinicians to provide better care for the best prognosis, through improved standardization and saving time via automation of repetitive tasks.

## Figures and Tables

**Table 1:** 2019 WHO classification of NENs.

Biology	Grade	Mitotic Count ^1^	Ki-67 Index ^2^
	G1	<2	<3
Well-Differentiated (NETs)	G2	2–20	3–20
	G3	>20	>20
Poorly Differentiated (NECs)	G3	>20	>20

^1^ Report number of mitotic figures in a 2 mm^2^ area. ^2^ Report percentage of Ki-67^+^ cells for 500–2000 cells.

**Table 2:** Histopathological grading and imaging breakdown. H&E-stained slides capture general tissue architecture and morphology and are used to ascertain mitotic count. Ki-67 staining is used to observe cells expressing the nuclear protein Ki-67, which is present in all actively dividing cells. These measures are central to NEN grading (see Table 1).

Stain	Unit		Grade	
		G1	G2	G3
H&E	Patients	109	56	21
	Slides	145	76	26
Ki-67	Patients	77	38	20
	Slides	78	38	22

**Table 3:** Three-fold classification of NEN grades for various methods and architectures.

Source	Method	3-Fold Average Balanced Accuracy (%)
H&E	Patch-Based	52.8
	DeepMIL [29]	46.6
	VarMIL [31]	36.5
	NoisyAND [32]	41.5
	Histogram MLP RetinaNet-DA [25]	77.5
H&E + Ki-67	Naïve Combination 1	82.1
	Naïve Combination 2	82.1
	**MLP Concatenated Features**	**83.0**
	Log Concatenated Features	74.5

Bold indicates best result.

## Data Availability

The MIDOG22 dataset is available for download on Zenodo.org [https://zenodo.org/records/4643381], accessed on 24 July 2023. Data from our internal dataset is available for collaboration upon written request of the authors.

## Abbreviations

The following abbreviations are used in this manuscript:

NEN	Neuroendocrine Neoplasm
H&E	Hematoxylin and Eosin
NEC	Neuroendocrine Carcinoma
WHO	World Health Organization
GI	Gastrointestinal
WSI	Whole Slide Image
MIDOG22	Mitosis Domain Generalization Challenge 2022
MIL	Multiple Instance Learning
MLP	Multi-Layer Perceptron
KM	Kaplan–Meier
